# Nurses' Diet at Six Shillings a Head

**Published:** 1912-08-24

**Authors:** 


### INSTITUTIONAL HOUSEKEEPING.

### Nurses' Diet at Six Shillings a Head.

The following dietary can be supplied for 6s. per week per head and will allow for everything provided to be of excellent quality: Meat, best foreign; eggs, new laid in summer, water-glass preserved in winter. The cost has been tested by an experienced matron who has planned it for her own household. An alternative (cold joint usually best) should be provided for dinner and supper, and a cold dish as alternative for breakfast. Unless the numbers are large, a variety of dishes will prove extravagant, as there will be many odds and ends over. When cold meat is to form the meal the joints should not be cut while hot. Separate joints should be cooked and allowed to cool. With good service such extras as toast, made sauces, salad dressings, etc., need not be expensive, but in the hands of the inexperienced they may lead to great extravagance, and be far more provocative of grumbling than their absence.

The meal provided for supper will often be quite suitable for the night nurses' ward meal. When it is not, eggs, with cake or stewed fruit, cold ham or sausages, with fresh fruit, etc., should be provided for the night nurses. A supply of tea, sugar, cocoa, and jam should be given out each week; butter at least twice a week to ensure freshness.

### A WEEK'S MEALS—SUMMER.

| | BREAKFAST. | DINNER. | SUPPER. |
|---|---|---|---|
| **Sunday:** | Eggs. Potted Meat. Marmalade. | Cold Roast Beef. Potatoes. Salad. Fruit Tart and Custard. | Cold Ham. Salad. Biscuits. Butter. Cheese. Coffee. |
| **Monday:** | Fish Cakes. Lettuce or Watercress. | Boiled Mutton, Caper Sauce. Two Vegetables. Blancmange. Jam. | Mince. Potatoes. Stewed Fruit. Cheese. Lemonade. |
| **Tuesday:** | Fried Bacon. Watercress. | Cold Steak Pies. Potatoes. Cucumber. Cornflour Pudding. Stewed Fruit. | Fish and Potatoes. Plain Cake. Cheese. Coffee. |
| **Wednesday:** | Sardines. Fruit. | Roast Mutton. Two Vegetables. Cold Tartlets. Fruit. | Spinach and Poached Eggs. Rice Pudding. Lemonade. |
| **Thursday:** | Ham. Marmalade. Lettuce. | Cold Salt Beef. Potatoes. Salad. Boiled Fruit Pudding. | Curry and Rice. Coffee. Cheese. Butter. Biscuits. |
| **Friday:** | Eggs (Poached). Boiled Bacon. | Fried Fish. Potatoes. Fruit Tarts. | Boiled Bacon, Beans and Parsley Sauce. Sago Pudding. Cheese. Coffee. |
| **Saturday:** | Fish Pie. Lettuce. | Cold Joints. Potatoes. Sauce. Pickles. Currant Batter Pudding. | Rissoles. Cheese. Coffee. Stewed Fruit. Custard. |
| | Tea or Coffee. Toast. Butter. Brown and White Bread. | *Alternative.* Cold Meat. Milk Pudding. | *Alternative.* Cold Meat or Soup. Milk instead of Coffee. |
| | TEA. Cake. Fruit. Jam on alternate days. | *Drinks.* Milk or Lemonade. Hot and Cold Water. Ice in hot weather. (Ale could be provided, but is rarely taken.) | |

### Housekeeping Queries.

Nurse Beta.—*Please suggest some convalescent dishes easy to make.*

Baked Omelet.—Separate the yolks and whites of three eggs; to the yolks add a level tablespoonful of flour and a saltspoonful of salt. Work these three together until the mixture looks creamy. Then stir in two tablespoonfuls of milk and the same amount of grated or very finely-chopped nuts. Whisk the whites of the eggs very stiffly and fold them lightly into the yolk mixture, and pour it all into a buttered *au gratin* or shallow pie-dish. Bake the omelet in a moderately hot oven for about ten to fifteen minutes, or until well puffed up, lightly browned, and spongy to the touch. Serve at once in the dish.

Cheese Puff is worth a trial. Melt an ounce of butter in a saucepan, stir smoothly into it one ounce of flour. Add half a pint of milk, and stir until boiling. Add three tablespoonfuls of grated cheese and the yolks of two eggs. Beat very thoroughly and season carefully. Next stir in lightly the whisked whites of the two eggs, and turn the mixture into a greased fireproof dish. Bake about fifteen minutes in rather a quick oven and serve at once. All light mixtures containing whipped white of egg need to be served at once or they soon sink down and become tough.

C. B.—*Can you give me a recipe for making gluten bread?*

Mix four ounces of almond meal with two tablespoonfuls of gluten meal and two teaspoonfuls of baking-powder. Warm one ounce of butter and work it well into the dry ingredients with enough salt to flavour agreeably. Add one raw egg, beat it well in, then add a second, and beat that in also. Turn the mixture into a shallow buttered tin, and bake about twenty-five minutes. Sometimes prepared bran is substituted for gluten meal.

Matron *writes: "I should be so glad of a plain suet pudding suitable for the staff dinner, one a little out of the common run if possible and yet very simple."*

Brown George Pudding is a universal favourite. Mix together ten ounces of flour, six ounces of chopped suet, two ounces of Demerara sugar, four ounces of stoned and halved raisins, and four ounces of currants. Well beat one egg, mix with it three tablespoonfuls of golden syrup or treacle, and pour these into the middle of the flour, etc. Mix one level teaspoonful of carbonate of soda with one gill of milk, pour these in and mix very thoroughly. Pour the mixture into a greased basin, cover the top with a piece of greased paper, and steam steadily for three hours. Turn out and pour some hot golden syrup round as sauce.

NOTICE TO OUR READERS.

The Editor welcomes for Institutional Housekeeping any contribution from those engaged in the work of the commissariat in institutions. All accepted contributions are paid for. Queries answered on any subject relating to the supply, cost, storage, distribution, or preparation of food.

